# Factors associated with early menarche: results from the French Health Behaviour in School-aged Children (HBSC) study

**DOI:** 10.1186/1471-2458-10-175

**Published:** 2010-03-30

**Authors:** Adrien Gaudineau, Virginie Ehlinger, Christophe Vayssiere, Beatrice Jouret, Catherine Arnaud, Emmanuelle Godeau

**Affiliations:** 1 UMR Inserm U558/University Paul Sabatier, Toulouse, France; 2 CHU Hautepierre/University Louis Pasteur, Strasbourg, France; 3 Maternity Paule de Viguier/University hospital, Toulouse, France; 4 Pediatric endocrinology departement/University hospital, Toulouse, France; 5 Clinical epidemiology departement/University hospital, Toulouse, France; 6 Service Médical du Rectorat, Toulouse, France

## Abstract

**Background:**

Puberty is a transition period making physiological development a challenge adolescents have to face. Early pubertal development could be associated with higher risks of poor health. Our objective was to examine risk behaviours, physical and psychological determinants associated with early menarche (<11 years).

**Methods:**

Early menarche was assessed in the Health Behaviour in School-aged Children French cross-sectional survey. Data were collected in 2006 by anonymous self-reported standardized questionnaire from a nationally representative sample of 1072 15 years old girls in school classrooms. Family environment, school experience, physical and psychological factors, risk behaviours (substance use and sexual initiation) were recorded. Logistic regression models were applied (analysing for crude and adjusted relationships between early menarche and risk behaviours controlled for family context).

**Results:**

Median age at menarche was 13.0 years; 57 girls (5.3%) were early-matured. Controlled for familial environment, early menarche was associated with having had more than two life-drunkenness episodes (adjusted OR = 2.5 [1.3-4.6]), early sexual initiation (adjusted OR = 2.8 [1.3-6.0]) and overweight (adjusted OR = 7.3 [3.6-14.9]).

**Conclusion:**

Early-maturing girls may affiliate with older adolescents, hence engage in risk behaviours linked to their appearance rather than their maturity level. Factors associated with early menarche highlight the need to focus attention on early-matured girls to prevent further health problems linked to risk behaviours.

## Background

Adolescence is a transition period from childhood to adult life during which pubertal development and sexual maturation take place. During puberty, hormonal, psychological, cognitive and physical changes occur simultaneously and interactively making physiological development a challenge adolescents have to face, with emotional, social and behavioural dimensions. A feature of sexual maturation in the human race is the 4 to 5 years physiological variation of pubertal age observed in normal individuals living in the same conditions [1]. This variability is mainly due to genetic, ethnic, environmental and nutritional factors [2]. Considering girls, age variations of menarche may be important: previous studies have shown that 5% of the population reported ages at onset before 10 or after 15 years [3]. Therefore, within a same biological age, variability exists not only in adolescent's physical appearances directly linked to hormonal effects, but more widely in their behaviours. Previous research has described that girls feeling "on-time" regarding their puberty had the most positive feelings in terms of pubertal development [4]. Therefore, in agreement with some authors [5], anomalies in pubertal timing (early or delayed pubertal development) could be associated with higher risks of poor health. Early pubertal development is of particular medical interest in terms of care management and prevention implications. Previous studies on factors associated with early pubertal development have focused their attention on one specific aspect of health only (whether in terms of family environment, physical or psychological factors or risk behaviours) despite the fact that all those factors interact to define global health as given by the World Health Organization (WHO).

The objective of this paper is to examine multiple factors (school experience, physical or psychological factors and risk behaviours) associated with early menarche in a large national representative sample of schoolgirls, using the French data from the 2006 Health Behaviour in School-aged Children (HSBC) international study.

## Methods

Data were obtained from the HBSC international WHO collaborative cross sectional study [6], carried out every four years since 1982. This survey aims to better understand health, well-being, health behaviours and social conditions of school children aged 11, 13 and 15 years and to examine self-perceived determinants of health. Records allow not only to examine the trend of health's indicators but also to initiate prevention strategies to promote youth's health. France has participated in 2006 for the fourth time [7], along with 40 other countries or regions sharing a common protocol and using the international standardized questionnaire [8] available in all the participating countries.

The French HBSC 2006 population included 7 154 pupils (3 558 boys and 3 596 girls) from the 8th to the 13th grade, sampled in public or private schools (school and pupils participation rates: 79.1 and 81.1%, respectively). Sampling strategy consisted in a stratified random design (stratification in four categories of school settings and seven educational levels) clustered into schools and classrooms. Because some risk behaviour variables were only asked of the oldest group (drugs consumption, sexual initiation), analyses were restricted to the 15 year-old girls (n = 1 072). We defined early menarche as a reported onset of menarche before 11 years. Depending on studies, early menarche is defined from 9 to 11.5 years [9-11]. This variability, though important, is explained by its relation with the distribution of the age at onset of menarche in the studied populations. Therefore, age limits to define early menarche are neither definitive nor universal. In this study, we hypothesized that anomalies in pubertal timing could be defined using the 5^th ^percentile of the observed distribution of age at menarche in our population.

The self anonymous classroom-administered questionnaire was developed by the HBSC international research network. All items were piloted and pre-tested at international and national levels prior to the main survey [12-15]. Parental consent was assessed. At students' level, participation in the survey was voluntary, with assurances provided in relation to confidentiality and anonymity. Age at menarche was derived from the following question: "Have you already had your periods?" with possible answers: "No, I haven't yet"/"Yes, I have at the age of: (year, month)".

Various factors associated to early menarche asked in the HBSC survey (family environment, school experience, physical and psychological factors, risk behaviours) were included in the analyses in accordance to the literature [6,16,17] and to our initial hypothesis that more mature physical appearance at the same biological age may be associated with higher risks of poor physical and psychological health and higher frequency of risk behaviours. Family environment variables were: family structure with response options of "traditional" (living with both biological father and mother) and "other family structure"; parental employment with "at least one parent working" and "none"; socio-economic status assessed by the Family Affluence Scale (FAS) [18] with "medium to high" and "low". School experience variables were: school results with "average to higher" and "below average"; liking school with "yes" and "no"; peers acceptance with "high" and "low". Risk behaviours variables were: daily smoking with "no" and "yes"; life drunkenness episodes with "<2" and "≥2"; life cannabis use with "<3 episodes" and "≥3 episodes"; sexual initiation before 15 years (colloquial terminology such as "having sex" or "going all the way" was added to ensure that respondents understood the question asked about full penetrative sex) with "no or not yet" and "yes". Physical and psychological factors included: Body Mass Index (BMI) (calculated from self-reported height and weight) with "normal or thin" and "overweight or obese". as recommended by the International Obesity Task Force [19]; body image perception with "normal or thin" and "overweight"; diet use with "no" and "yes"; self-rated health with "good" and "bad"; life assessment from the Cantril scale [20] with "high" and "low"; recurrent health complaints [6] (defined as two symptoms or more at least once a week, from which depression, irritability, nervousness, headaches, stomach-aches, back-aches, dizziness and sleeping troubles) with "no" and "yes".

Because pubertal development was not achieved in all girls taking part in the study, survival analysis methods were used to estimate the distribution of age at menarche (Kaplan-Meier). Mean and standard deviation (SD) was also reported in the 15 year-old girls who had already experienced menarche in order to compare our data to other studies. We compared frequencies of exposition to each predefined variables according to menarche status (early versus others) using a χ^2 ^test and a Fisher's exact test where needed. We then performed a multivariable logistic regression analysis to determine factors associated with early menarche. School experience, risk behaviours, physical and psychological factors were included in the initial model as covariates if they were related to early menarche at a 20% significance level in the univariate analysis. A backward procedure was then used to remove variables from the model (1% significance level), adjusting for familial environment characteristics. Regressions excluded girls who had missing values on any of the variables considered in the initial model. The final model was rerun using all available observations to check stability. All the analyses took into account the sampling strategy, setting classes as primary sampling units. Adjusted and unadjusted odds ratios (ORs) and 95% confidence intervals (CIs) are reported. All analyses were performed using STATA statistical package (STATA v9.0 College Station, TX) [21] and took into account the sampling strategy.

The study protocol was approved by the Ministry of Education and the French National Commission of Computer Science and Freedom, as the national review board for surveys involving people and data management.

## Results

Median and mean age at menarche were 13.0 years (interquartile range (IQR) 12.0-13.8) and 12.8 years (SD 1.2) respectively. In total 57 girls were considered early-matured (5.3% in our 15 year-old population).

Factors associated with early menarche are shown in table 1. Frequency of girls living in a non-traditional family structure was significantly greater among early-matured girls than among others (45.6% versus 27.1%), as well as frequencies of low peers acceptance (42.1% versus 25.3%), having had more than two life drunkenness episodes (36.4% versus 17.6%), early sexual initiation (28.9% versus 11.1%), poor health perception (35.1% versus 20.2%), recurrent health complaints (71.9% versus 51.7%). Frequencies of girls either overweight according to self-reported BMI (29.1% versus 7.0%) or with an overweight body image (63.2% versus 42.9%), and weight loss diet use (31.6% versus 17.9%) were again higher among early-matured girls than among others.

**Table 1:** Factors associated to early menarche in 1 072 girls aged 15 years. Univariate analysis.

	Early menarche (N = 57) n (%)	**Menarche ≥ 11 y**. (N = 1 015) n (%)	OR (95%CI)	p value*
**Familial environment**				
Family structure				
traditional	31 (54.4)	739 (72.9)	1	0.004
other family structure	26 (45.6)	275 (27.1)	2.3 (1.3-3.9)	
Parental employment				
at least one parent working	54 (94.7)	937 (92.9)	1	0.590
none	3 (5.3)	72 (7.1)	1.4 (0.4-4.5)	
FAS				
medium to high	43 (76.8)	881 (87.4)	1	0.026
low	13 (23.2)	127 (12.6)	2.1 (1.1-4.0)	
**School experience**				
School results				
average to higher	42 (73.7)	815 (80.6)	1	0.186
below average	15 (26.3)	196 (19.4)	1.5 (0.8-2.7)	
Liking school				
yes	32 (58.2)	605 (59.8)	1	0.812
no	23 (41.8)	407 (40.2)	1.1 (0.6-1.9)	
Peers acceptance				
high	33 (57.9)	754 (74.7)	1	0.008
low	24 (42.1)	255 (25.3)	2.2 (1.2-3.8)	
**Risk behaviours**				
Daily smoking				
no	39 (76.5)	816 (84.0)	1	0.139
yes	12 (23.5)	155 (16.0)	1.6 (0.9-3.1)	
Life drunkenness episodes				
<2	35 (63.6)	810 (82.4)	1	0.001
≥2	20 (36.4)	173 (17.6)	2.7 (1.5-4.6)	
Life cannabis use				
<3 episodes	44 (78.6)	852 (86.5)	1	0.108
≥3 episodes	12 (21.4)	133 (13.5)	1.8 (0.9-3.5)	
Sexual initiation <15 years				
no or not yet	37 (71.1)	856 (88.9)	1	0.001
yes	15 (28.9)	107 (11.1)	3.2 (1.7-6.3)	
**Physical and psychological factors**				
Self-reported BMI				
normal or thin	39 (70.9)	911 (93.0)	1	<0.001
overweight or obese	16 (29.1)	68 (7.0)	5.5 (2.9-10.6)	
Body image				
normal or thin	21 (36.8)	579 (57.1)	1	0.004
overweight or obese	36 (63.2)	435 (42.9)	2.3 (1.3-4.0)	
Diet use				
no	39 (68.4)	825 (82.1)	1	0.023
yes	18 (31.6)	180 (17.9)	2.1 (1.1-4.0)	
Self-rated health				
good	37 (64.9)	807 (79.8)	1	0.011
bad	20 (35.1)	204 (20.2)	2.1 (1.2-3.8)	
Life assessment				
high	39 (68.4)	781 (77.4)	1	0.137
low	18 (31.6)	228 (22.6)	1.6 (0.9-2.9)	
Recurrent health complaints				
no	16 (28.1)	489 (48.3)	1	0.005
yes	41 (71.9)	524 (51.7)	2.4 (1.3-4.4)	

Abbreviations: FAS = Family Affluence Scale; BMI = Body Mass Index; OR = Odds Ratio; CI = Confidence Interval

*χ^2^-test or Fisher's exact test where necessary

All analysis took into account the sampling strategy

The final multivariate model showed that having had more than two life drunkenness episodes, a sexual initiation before 15 years and being overweighted or obese remained significantly and independently associated with early menarche (p < 0.01), after controlling for familial environment (i.e. family structure, parental employment and FAS) (table 2).

**Table 2:** Factors associated to early menarche in 956 girls aged 15 years. Multivariable logistic regression analysis.

		Adjusted OR (95%CI)	p-value
**Life drunkenness episodes**	≥ 2	2.5 (1.3-4.6)	0.006
**Sexual initiation**	< 15 years	2.8 (1.3-6.0)	0.007
**BMI**	overweight or obese	7.3 (3.6-14.9)	< 0.001

Abbreviations: FAS = Family Affluence Scale; BMI = Body Mass Index; OR = Odds Ratio; CI = Confidence Interval

All analysis took into account the sampling strategy and controlled for familial environment (family structure, parental employment, FAS).

## Discussion

Age at menarche has largely decreased in most developed countries and seems stabilised at 13 years with 0.5 years variations between countries [22]. Our results are in line with this trend. According to some research [23], decrease in mean age of menarche could be associated with a decrease in its variability (as measured by standard deviation). With 1.2 years, our standard deviation is narrower than in previous studies on the same population. Since girls with an early menarche belong to a deviant category from peers, even more when age at onset is homogeneous [5], our finding is important because of its potential impact on early-matured girls' behaviours.

From our results, after controlling for familial environment, to have had more than two life drunkenness episodes, a sexual initiation before 15 years and self-reported overweight or obesity remained the only factors significantly and independently associated with early menarche (p < 0.01). Health risk behaviours are major contributors to morbidity and mortality among adolescents [24]. Although initiation of some of these behaviours in middle or late adolescence may reflect psychologically healthy experimentation [25], adolescents who initiate health risk behaviours at an early age appear to be at greater risk for negative consequences later in life [26]. The mediating mechanisms at stake to understand the association between risk behaviours and early menarche is still debated in the literature. According to some authors, early pubertal development could result in affiliation with older adolescents, who often experience increased deviance and substance use [27]. Therefore, affiliation with an older peer group putatively increases the risk for initiation and addiction because of greater availability of substances, peer modelling of use and biased perceptions of substance use norms. Early-matured girls may therefore face pressure to engage in behaviours appropriate to their appearance rather than their experience, coping or cognitive abilities [28]. For others, early pubertal development could lead to depression and more frequent risk behaviours [11,17,24,28]. From our results, we cannot conclude on this hypothesis: though the association of lower peer acceptance with early menarche is significant in univariate analysis (table 1), the cross-sectional methodology with self-perceived determinants of health is submitted to various biases.

In Stice et al's prospective study of 496 girls [11], early menarche (defined as an age at onset of menarche before 11.6 years) was associated with increased substance use and abuse (defined as two or more impairments of role obligations directly resulting from substance use). Although in line with this association, our results showed that among all substance use variables tested (tobacco, cannabis, alcohol), only to have had two or more life drunkenness episodes was associated with early menarche (adjusted OR = 2.5 95%CI [1.3-4.6]).

Drunkenness is an indicator of alcohol misuse. Frequent or heavy alcohol users report poorer subjective and overall health and a greater number of overnight hospital stays than infrequent or non users [29]. There is a strong association between adolescent alcohol misuse and an array of other behaviours or conditions, such as smoking and illegal drug use, risky sexual behaviour, disruptive behaviour, depressive and anxiety disorders, eating disorders and obesity [29]. There is evidence that these behaviours cluster in young people with high-risk lifestyles [29]. The association we found could in fact be even stronger than shown because based on self-perceived determinants of health, leading to a risk of understatement although our questionnaire was validated from previous HBSC studies in the same context, and the lack of power of our analysis (by definition, early menarche was rare).

The second type of risk behaviour included in our model and significantly associated with early menarche was early sexual intercourse (defined as ever having had sexual intercourse before 15 years). A 3-4 years decrease of age at first intercourse was observed in Western countries during the last half of the XX^th ^century [30]. Early sexual initiation could have negative effects on health due to the developmental inabilities to deal with consequences of such sexual activity. Studies have shown that it was associated with a lesser use of contraceptive methods and an increase in sexual transmitted diseases exposure [31].

Furthermore, several research findings suggest that early sexual activity more likely reflects problems in adolescent development than successful rite of passage [32]: thus it has been found associated with other risk behaviours such as smoking tobacco, higher levels of drunkenness and cannabis use and frequent evenings out with friends [33]. In addition, early sexual initiation has been associated with poorer health-related quality of life among girls [33]. In our study, sexual initiation before 15 years was about three times more frequent for early-matured girls compared to others (adjusted OR = 2.8 95%CI [1.3-6.0]).

Even if our study was cross-sectional by design, we can hypothesise that those two risk behaviours are probably consequences of early menarche (only one girl had sexual intercourse and three had been drunk more than twice before 11 years).

Potential physical influences of age at menarche include factors such as weight, nutrition and exercise [34]. The World Health Organization recognizes that childhood overweight and obesity have reached epidemic proportions in most industrialized countries. BMI is associated with direct measures of fatness, cardiovascular risk factors, social and psychological problems and with poor general health-related quality of life [35]. A high BMI during childhood and adolescence is associated with an increased risk of adult obesity [35] and premature mortality [36]. In our study and as hypothesized, self-reported overweight or obesity remained strongly associated with early menarche (adjusted OR = 7.3 95%CI [3.6-14.9]). However, due to the cross-sectional design of our study, direction of this association cannot be known. In the literature, this question is still debated [37]: on the one hand, girls who mature early tend to be more frequently obese as adults (oestrogens promoting deposition of fat in peripheral adipose tissue) [38]; on the other, childhood BMI is associated with an earlier menarche (due to oestrogens' production by peripheral adipose tissue). These findings support the need to explore such associations in longitudinal studies.

To eliminate potential biases, we controlled our results for familial environment factors because individual differences in the timing of pubertal maturation are influenced by both genes and environment. According to Belsky et al [16], early development may be environmentally triggered and may actually be an adaptive response to a stressful environment (marital conflict, father absence, poor parenting or low socio-economic status). Though controversial, the Belsky et al.'s theory has been supported by other studies [17]. A potential limitation of our study lies in our choice of pubertal development estimate: the first manifestation of puberty is the development of the mammal gland. Menarche appears 2 to 2.5 years after the beginning of puberty, but timing is shortened in the case of late puberty. However, age at menarche, because easy to determine and to memorise, serves as an estimate in many studies [39]. Information on age at menarche is often retrospectively identified in most studies [40], hypothesising that menarche is a memorable event in a woman's life. Previous studies have shown a good correlation between stated and real age at menarche within a 4-year-recall period [40]. Because in our study, girl's ages range from 14.6 to 16.4 years, we made the assumption that the memory bias could be neglected.

## Conclusions

In conclusion, our results add to a growing literature [41] that suggests that girls who experienced early menarche are more likely to engage in risk behaviours such as alcohol abuse and early sexual initiation. These results highlight the need for more awareness and more prevention information in the case of early menarche and the overall need to reinforce prevention around puberty. More research is needed to better understand the associations between risk behaviours and early menarche on larger populations from collaborative studies, and furthermore on how obesity interacts with early menarche and risk behaviours.

## Competing interests

The authors declare that they have no competing interests.

## Authors' contributions

AG analysed the data and wrote the manuscript. VE contributed to the analysis. CV & BJ have made substantial contributions to interpretation of data. CA & EG conceived of the study, contributed to the analysis and co-wrote the manuscript. All Authors read and approved the final manuscript.

## Pre-publication history

The pre-publication history for this paper can be accessed here:

http://www.biomedcentral.com/1471-2458/10/175/prepub
